# Sensitisation to peanut LTP (rAra h 9) in children allergic to peach

**DOI:** 10.1186/2045-7022-5-S3-P131

**Published:** 2015-03-30

**Authors:** Teresa Boyano-Martinez, Maria Pedrosa, Carmen Garcia-Ara, Santiago Quirce

**Affiliations:** 1 Hospital Universitario La Paz, Madrid, Spain

## Introduction

Non-specific lipid transfer proteins (LTPs) are panallergens present in plant foods and are recognized as maior allergens in patients allergic to Rosaceae family fruits from the Mediterranean area. On the contrary, seed storage proteins are the most important allergens in peanut allergy worldwide, although some authors from Southern Europe found Ara h 9 (peanut LTP) as the maior allergen. The aim of the study was to analyze the sensitization to Ara h 9 in children allergic to peach, and the association with clinical tolerance to peanut.

## Patients and methods

Thirty-three children with clinical history of peach allergy and specific IgE antibodies to Pru p 3 were included. Seventeen children allergic to peanut were used as the control group. Specific IgE to peanut extract, nPru p 3, rAra h 9 and rAra h 2 were determined by ImmunoCAP (Thermo Fisher). Titers higher than 0.34 KUA/L were considered positive. The clinical tolerance to peanut was evaluated by a questionnaire administered by the allergist.

## Results

**Figure 1:**
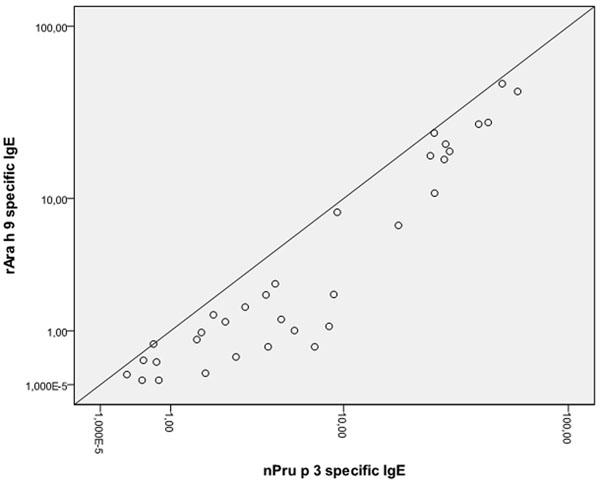
Correlation between nPru p 3 and rAra h 9 levels (kU/L).

The mean age of the study group was 6.7 years (SD 3.4), and the relation male/female 24/26. Twenty-four (72.7%) children allergic to peach had positive specific IgE antibodies to peanut extract and 28 (84.8%) to rAra h 9. There was a high association between nPru p 3- and rArah 9-specific IgE levels (R2=0.925; p<0.001)(Fig. 1). In this group 16 children (48.5%) referred good tolerance to peanut ingestion, 10 (30.3%) had never eaten it, and 7 (21.2%) had suffered allergic reactions related with the nut.

In the peanut allergic group 14 (82.4%) children had positive IgE antibodies to rAra h 2. Only 3 (17.6%) children had specific IgE to rAra h 9, and all of them had also IgE to rAra h 2.

## Conclusions

The majority of children sensitized to Pru p 3 had specific IgE antibodies to Ara h 9 and to the whole peanut extract. At least a half of them can eat peanut with good tolerance. On the contrary, children allergic to peanut recognized more frequently Ara h 2.

